# Management of pancreatic arteriovenous malformation: Case report and literature review

**DOI:** 10.1097/MD.0000000000027983

**Published:** 2021-12-23

**Authors:** Wei Wu, Feng-Duo An, Cheng-Lin Piao, Ming-Kun Tan, Zhen-Duo Si, Lan Xin, Na Zhao, Jian-Jun Leng

**Affiliations:** Hepatopancreatobiliary Surgery, Peking University Shougang Hospital, Beijing, China.

**Keywords:** case report, gastrointestinal hemorrhage, pancreatic arteriovenous malformation, pancreaticoduodenectomy, transarterial embolization

## Abstract

**Introduction:**

Pancreatic arteriovenous malformation (P-AVM) is a rare vascular malformation. Fewer than 200 cases have been reported. The clinical manifestations lack specificity. Common symptoms include abdominal pain, gastrointestinal hemorrhage, and jaundice, which is easily confused with other disorders.

**Patient concerns:**

A 42-year-old man received TAE due to abdominal pain caused by P-AVM in a local hospital, melena and abdominal pain occurred in a short time after TAE.

**Diagnosis:**

The patient was diagnosed as P-AVM which was confirmed by computed tomography and digital subtraction angiography.

**Interventions:**

A pylorus-preserving pancreatoduodenectomy was successfully performed after diagnosis was made.

**Outcomes:**

The patient recovered with no complications two weeks after surgery, and no sign of recurrence was found during the 4-mo follow-up period.

**Conclusion:**

In our experience, TAE may have limitations in the treatment of P-AVM and surgical resection should be considered as the treatment of choice.

## 1 Introduction

Pancreatic arteriovenous malformation (P-AVM) is a rare disease with a low incidence.^[1]^ To our knowledge, few cases of P-AVM have been reported to date. The presentation of P-AVM is usually nonspecific, and common symptoms include abdominal pain, gastrointestinal hemorrhage and jaundice. The main therapeutic approaches include surgical resection and transarterial embolization (TAE).^[1]^ We report a 42-year-old man with AVM in the head of the pancreas who had a relapse of symptoms after TAE. This case provides clinical strategies for the diagnosis and treatment of P-AVM.

## 2 Case presentation

### 2.1 Chief complaints

A 42-year-old man with intermittent upper abdominal pain for 2 years and melena for >1 month was sent to the Emergency Department of our hospital.

### 2.2 History of present illness

In 2018, the patient presented with abdominal pain without any recognizable precipitating factors and was admitted to a local hospital. Enhanced abdominal computed tomography (CT) (November 2018) suggested multiple fistulas in the intrapancreatic-portal system with P-AVM. Following symptomatic treatment for pain relief, his symptoms were relieved and he did not receive further treatment at that time. Shortly after this, the patient presented with recurrent abdominal pain, and available symptomatic treatments relieved his clinical manifestations.

In September 2020, the patient received gastroduodenal artery TAE in a local hospital, and although the preoperative symptom of abdominal pain was relieved, intermittent melena (10 mL/day) associated with fever occurred after embolization. Re-examination with abdominal CT scans (October 2, 2020, in a local hospital) showed post-embolization changes of the AVM, and multiple low-density lesions were observed in the tail of the pancreas and spleen. One month after embolization (November 12, 2020), the patient revisited the hospital with acute abdominal pain, fever, nausea, and hematemesis (the amount of blood loss was approximately 10 mL) and he was admitted in our Emergency Department.

### 2.3 History of past illness

The patient had a history of hypertension treated for 20 years.

### 2.4 Physical examination

Abdominal examination revealed tenderness in the epigastric area and no masses were palpable.

### 2.5 Laboratory examinations

Routine blood test showed anemia with a hemoglobin level of 7.3 g/mL, a slight elevation in serum pancreatic enzymes (149 IU/L), and a positive fecal occult blood test was observed.

### 2.6 Imaging examinations

**Figure 1:**
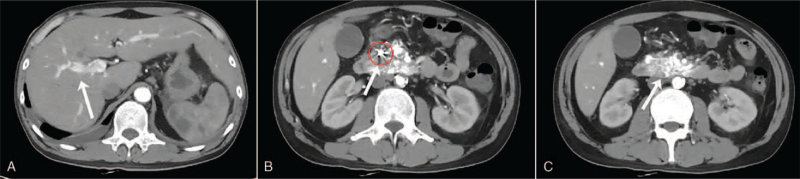
Enhanced abdominal CT findings. Enhanced abdominal CT indicating that the portal vein was filled early with arterial blood during the arterial phase (A), and dot-like small vessels with intense signals in the lesion of the pancreatic head were observed (B). A coil-like metal shadow was seen in a branching vessel of the gastroduodenal artery (read cycle) (C).

**Figure 2:**
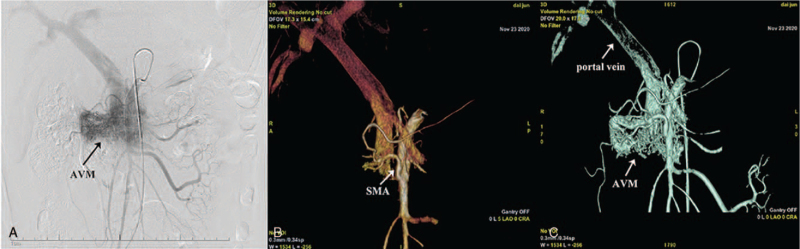
Digital subtraction angiography findings. Digital subtraction angiography showing multiple tortuous vascular shadows in the head of the pancreas, consistent with AVM (A). The superior mesenteric artery is the main supply artery (B). 3D reconstruction model (C).

A new enhanced CT scan (November 18, 2020 at our hospital) showed an AVM in the head of the pancreas associated with arteriovenous fistulas, and the supply arteries originated from the superior mesenteric artery, and drained directly into the splenic and portal vein through the arteriovenous fistula in the early arterial phase (Fig. 1A and B). A coil-like metal shadow was also seen in the gastroduodenal artery (Fig. 1C). Digital subtraction angiography (DSA) was performed under local anesthesia which revealed abnormal branch vessels of the superior mesenteric artery at the head of the pancreas which drained directly into the portal vein in the form of an arteriovenous fistula (Fig. 2A–C). Endovascular coils were visible in the gastroduodenal artery.

### 2.7 Treatment

Based on these findings, a diagnosis of P-AVM with arteriovenous fistula was made. After multidisciplinary team consultation, a standard pylorus-preserving pancreaticoduodenectomy was performed on December 14, 2020. After surgery, the patient received rehydration, infection prevention, and nutrition support.

### 2.8 Outcome and follow-up

**Figure 3:**
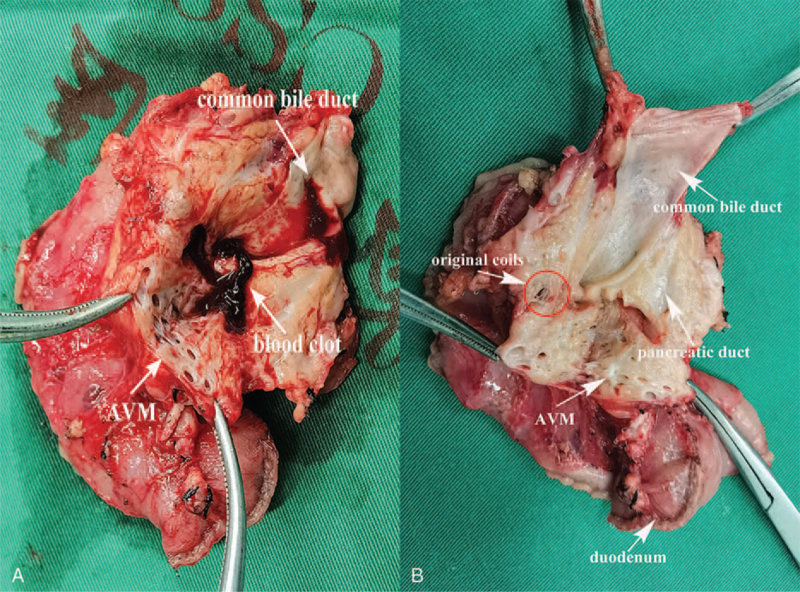
Postoperative pathology (gross specimen). Dissection of the pancreatic head shows malformed blood vessels in the head of the pancreas, blood clots in the pancreatic duct, and pancreatic duct dilation (A). The specimen after cleaning: original coil placement position can be seen (B).

**Figure 4:**
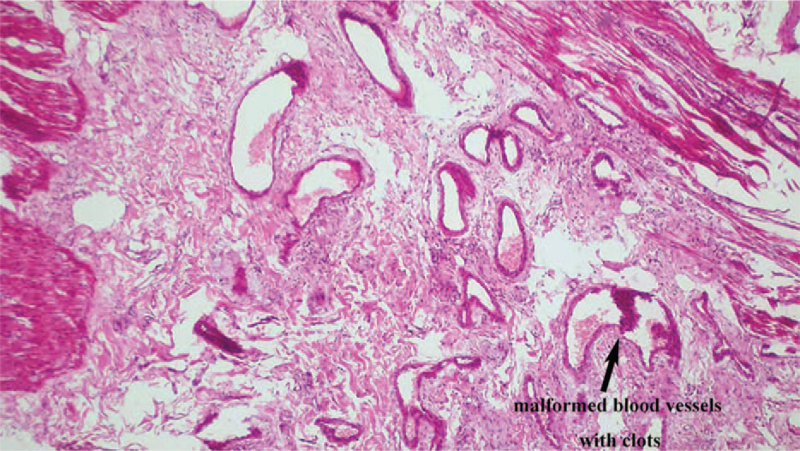
Postoperative pathology (under microscope). Deformed blood vessels of different thickness, and blood clots were seen in the vessels.

The patient stated that the symptoms improved remarkably at the postoperative period, re-examination of fecal occult blood was negative 1 week after the operation. Abdominal CT re-examination showed no signs of AVMs in the preserved pancreatic body and tail. Fourteen days after the procedure, the patient had recovered and was discharged without complications. Postoperative analysis of the surgical specimen revealed multiple dilated malformed blood vessels in the head of the pancreas and blood clots in the dilated pancreatic duct (Fig. 3). Microscopically, a large number of dilated and twisted blood vessels were observed in the pancreatic parenchyma, between the pancreas and duodenum and under the duodenal mucosa. The vascular lumens varied in size with congestion in part of the vascular cavity, consistent with AVMs (Fig. 4). No sign of recurrence was found by CT scan during the 4-month follow-up period.

## 3 Discussion and conclusion

P-AVM is characterized by malformations or telangiectatic lesions in the normal pancreatic mucosa or submucosal blood vessels, which result in excessive arterial blood draining into the portal venous system.^[1,2]^ Halper et al first described P-AVM in 1968, and since then <200 cases have been reported in the literature. Present research suggests that approximately 90% of patients with P-AVMs are caused by congenital disorders, which result from aberrant formation of the arteriovenous plexus during embryonic development. Approximately 10% to 30% of cases are associated with Osler-Rendu-Weber syndrome, an autosomal dominant inherited disorder.^[2,3]^

The clinical manifestations of P-AVM are usually nonspecific, and common symptoms include abdominal pain, melena, hematemesis, and jaundice, and some patients do not even have significant clinical symptoms.^[4]^ Chou et al^[5]^ reported the clinical manifestations caused by P-AVM in different parts of the pancreas, where hemorrhage is the most common presentation of P-AVM in the head of the pancreas, whereas pancreatitis often occurs in the tail. Almost 11% of patients will eventually develop portal hypertension or liver cirrhosis.^[6]^ In this case, the patient initially presented with abdominal pain after TAE; however, symptoms such as melena and hematemesis appeared. The literature shows that rupture of P-AVM may lead to hemorrhage, the blood enters the gastrointestinal tract through the pancreatic duct and bile duct, which may cause gastrointestinal bleeding.^[7,8]^ Abdominal pain may be related to the Steal syndrome caused by vascular embolization.^[9]^

In terms of diagnosis, color Doppler ultrasonography is helpful, the characteristic “mosaic sign” of vascular malformation can be observed in typical cases.^[1]^ On an enhanced CT scan, P-AVM displays an aggregation of small hypervascular spots in the pancreas, with a characteristic early fill of the portal vein or splenic vein during the arterial phase.^[2,10]^ DSA is the criterion standard for the diagnosis of P-AVM, and typical features include a high-density, twisted vascular network of abnormal blood vessels under fluoroscopy, and high-pressure arterial blood flow through the venous system during the early arterial phase.^[7,9]^

Existing studies show that the safety and therapeutic efficacy of surgical resection is superior to other treatment modalities, particularly in patients without portal hypertension, and surgical resection deserves consideration as the first treatment option.^[1,5]^ AVM in the pancreatic head may form vascular networks under duodenal mucosa, and we recommend that these patients undergo pylorus-preserving pancreatoduodenectomy. TAE, transjugular intrahepatic portosystemic shunt (TIPS), and radiotherapy may be nonsurgical approaches for patients with portal hypertension or who decline surgery.^[11–14]^ In our case, the patient had abdominal pain as the first presenting symptom. Although he experienced pain relief after embolization of a branch of the gastroduodenal artery, melena appeared and abdominal pain recurred within a short time. We speculate that pain relief may be related to a decrease in blood perfusion pressure in the head of the pancreas and a decrease in pancreatic capsule tension after TAE. According to the literature, the main reason for the failure of conservative treatments, such as TAE, is that the multiple malformed blood vessels cannot be completely embolized; in addition, new collateral circulation can be formed between these vessels, and eventually leads to recurrence.^[1,5,7]^ The patient underwent pylorus-preserving pancreaticoduodenectomy, and his symptoms completely disappeared after this procedure. The surgical specimen in this case also illustrated that it is difficult to achieve embolization of all abnormal blood vessels with TAE, which results in gastrointestinal bleeding and recurrent abdominal pain.

We analyzed relevant published cases treated by TAE in PubMed database from 1968 to 2020. The key words based on searches included “pancreas” and “arteriovenous malformation.” In total, 26 articles contained TAE as a therapy for P-AVM.^[1–3,11–32]^ The clinical characteristics and further treatment of 26 cases are summarized in Tables 1 and 2. The average age at diagnosis was 51.5 years (range 26 years to 67 years) and 24 of the 26 patients were male, 2 were female. The majority of these patients were from Asia (69.2%), with most cases reported in Japan (12 cases). The proportion is significantly higher than in Europe (15.4%, 4 cases), North America (11.5%, 3 cases) and South America (3.9%, 1 case). Abdominal pain (65.4%, 17 cases) and GI Bleeding (23.1%, 6 cases) were the main reasons for seeking medical care. Among these cases, 19 patients (73.1%) had a P-AVM located in the head of pancreas, 6 patients (23.1%) in the body and tail, and 1 patient (3.8%) in the head and body. Of the 26 patients who received TAE treatment, 12 (46.2%) had no significant clinical symptoms or complications, 6 (23.1%) had gastrointestinal bleeding after embolizing feeding arteries, 5 (19.2%) did not report the relevant contents in the literature, 2 (7.7%) found duodenal ulcers by gastroscopy after embolization, 1 case reported mild abdominal pain. 15 (57.7%) patients were successfully treated with TAE. Among them, 12 patients were completely cured without complications, 2 patients underwent surgical resection after embolization to control bleeding, and 1 patient had mild abdominal pain after embolization, which was considered unrelated to embolization. Of the 11 (42.3%) patients in whom P-AVM was not cured by TAE, rebleeding after embolization is the major reason for treatment failure. P-AVM usually has several supplying arteries, which are difficult to be embolised completely.^[1]^ TAE could be used as a definitive therapy mainly for P-AVM with a single feeding artery.^[1]^ Regarding the treatment after embolization, 10 patients (38.5%) underwent surgical resection; all these patients recovered well with no significant complications or recurrence. Of the remaining 3 patients, 2 (7.7%) received radiation and 1 (3.8%) underwent TIPS.

**Table 1:** Summary of cases diagnosed as P-AVM treated by TAE.

No.	References	Year	Age	Sex	Ethnic	Initial main symptoms	Location	Symptoms or complications after TAE	Treatment process
1	15	1982	52	Male	USA	Abdominal pain	Head	GI Bleeding	TAE→PD
2	16	1991	60	Male	Japan	Asymptomatic	Body or/ and tail	None	TAE
3	17	1993	66	Male	Japan	Abdominal pain	Head	None	TAE
4	18	1995	67	Male	Japan	Asymptomatic	Body or/ and tail	Unknown	TAE→Radiation
5	12	1998	45	Male	Japan	GI Bleeding	Head	GI Bleeding	TAE→TIPS
6	19	1999	48	Male	Japan	Abdominal pain	Head	Unknown	TAE→PPPD
7	20	2002	58	Male	Japan	GI Bleeding	Head	None	TAE
8	14	2003	60	Male	Japan	GI Bleeding	Head	GI Bleeding	TAE→Radiation
9	21	2006	45	Male	Japan	GI Bleeding	Head	Unknown	TAE→PPPD
10	3	2009	54	Male	Japan	Asymptomatic	Body or/ and tail	None	TAE
11	22	2010	55	Male	France	Abdominal pain	Head	None	TAE
12	23	2011	64	Female	Greece	Abdominal pain	Body or/ and tail	Abdominal pain	TAE
13	2	2011	26	Male	India	Abdominal pain	Head	GI Bleeding	TAE→PD
14	24	2011	47	Female	USA	GI Bleeding	Head	GI Bleeding	TAE→PPPD
15	25	2012	48	Male	Italy	GI Bleeding	Head	Duodenal ulcers	TAE
16	1	2012	46	Male	Korea	Abdominal pain	Head	Unknown	TAE→PPPD
17	1	2012	46	Male	Korea	Abdominal pain	Head	Unknown	TAE→PPPD
18	26	2013	37	Male	India	Abdominal pain	Head	GI Bleeding	TAE→PD
19	27	2014	49	Male	Japan	Abdominal pain	Head	None	TAE
20	11	2014	57	Male	Japan	Abdominal pain	Head	None	TAE
21	28	2015	56	Male	France	Abdominal pain	Head	Duodenal ulcers	TAE
22	29	2015	50	Male	Japan	Abdominal pain	Head	None	TAE→PPPD
23	30	2016	54	Male	USA	Abdominal pain	Head and body	None	TAE
24	31	2017	46	Male	Argentina	Abdominal pain	Body or/ and tail	None	TAE
25	32	2018	60	Male	India	Abdominal pain	Head	None	TAE→PPPD
26	13	2020	43	Male	Korea	Abdominal pain	Body or/ and tail	None	TAE

GI = gastrointestinal, P-AVM = pancreatic arteriovenous malformation, PD = pancreatoduodenectomy, PPPD = pylorus-preserving pancreaticoduodenectomy, TAE = transarterial embolization.

**Table 2:** Clinical characteristics of the patients with P-AVM treated by TAE.

	N = 26
Age, y, average (range)	51.5 (26–67)
Sex, n (%)	
Male	24 (92.3)
Female	2 (7.7)
Ethnic, n (%)	
Asia	18 (69.2)
Europe	4 (15.4)
North America	3 (11.5)
South America	1 (3.9)
Initial main symptoms, n (%)	
Asymptomatic	3 (11.5)
Abdominal pain	17 (65.4)
GI Bleeding	6 (23.1)
Location of P-AVM, n (%)	
Head	19 (73.1)
Body or/ and tail	6 (23.1)
Head and body	1 (3.8)
Symptoms or complications after TAE, n (%)	
GI Bleeding	6 (23.1)
Abdominal pain	1 (3.8)
Duodenal ulcers	2 (7.7)
None	12 (46.2)
Unknown	5 (19.2)
The result of TAE, n (%)	
Success^∗^^,^^†^	15 (57.7)
Failure	11 (42.3)
Treatment after TAE, n (%)	
None	13 (50.0)
Surgery (PD and PPPD)^‡^	10 (38.5)
PD	3
PPPD	7
Radiation	2 (7.7)
TIPS	1 (3.8)

GI = gastrointestinal, P-AVM = pancreatic arteriovenous malformation, PD = pancreatoduodenectomy, PPPD = pylorus-preserving pancreaticoduodenectomy, TAE = transarterial embolization, TIPS = transjugular intrahepatic portosystemic shunt.

∗ Two patients were asymptomatic but founded duodenal ulcers by endoscopy after TAE, they did not receive any other treatments for P-AVM.

† One patient reported mild abdominal pain after TAE, but did not receive further treatment for P-AVM.

‡ Two patients underwent surgery resection after bleeding vessels were embolized by TAE.

In conclusion, P-AVM is a rare pancreatic vascular disease. Common clinical manifestations include abdominal pain and gastrointestinal hemorrhage. With regard to diagnosis, color Doppler ultrasound can reveal the blood flow of malformed vessels in the lesions. Abdominal enhanced CT and DSA can help confirm the diagnosis. From our experience, surgical resection is the most effective treatment and pylorus-preserving pancreatoduodenectomy is the first choice for patients with AVM in the head of the pancreas. Other treatments such as TAE, TIPS, and radiotherapy do not completely eliminate the complications caused by P-AVM, and have limitations.

## Acknowledgments

The authors thank all nurses in our department for their dedicated care of the patient, and the pathologists in our hospital for their work on immunohistochemistry.

## Author contributions

Leng JJ guided the entire treatment and report writing;

Wu W wrote the report;

Leng JJ, An FD and Tan MK performed the surgery;

Piao CL, Wu W, Si ZD, Lan X and Zhao N contributed to the literature search;

All authors have read and approved the final version of the manuscript.

**Formal analysis:** Wei Wu, Mingkun Tan, Zhenduo Si, Lan Xin.

**Funding acquisition:** Wei Wu, Na Zhao.

**Investigation:** Fengduo An, Chenglin Piao.

**Project administration:** Jianjun Leng.

**Supervision:** Jianjun Leng.

**Writing – original draft:** Wei Wu, Jianjun Leng.

**Writing – review & editing:** Wei Wu, Fengduo An, Chenglin Piao.
